# Flaxseed oil increases aortic reactivity to phenylephrine through reactive oxygen species and the cyclooxygenase-2 pathway in rats

**DOI:** 10.1186/1476-511X-13-107

**Published:** 2014-07-03

**Authors:** Dieli Oliveira Nunes, Camila Cruz Pereira Almenara, Gilson Brás Broseghini-Filho, Marito Afonso Sousa Costa Silva, Ivanita Stefanon, Dalton V Vassallo, Alessandra S Padilha

**Affiliations:** 1 Department of Physiological Sciences, Federal University of Espirito Santo, Av. Marechal Campos, 1468, Maruípe, 29040-091 Vitória, ES, Brazil; 2 Health Science Center of Vitoria - EMESCAM, Vitoria, Espirito Santo, Brazil

**Keywords:** Omega-3, Flaxseed oil, Aorta, Vascular reactivity, Oxidative stress

## Abstract

**Background:**

Flaxseed oil has the highest concentration of omega-3 α-linolenic acid, which has been associated with cardiovascular benefit. However, the mechanism underlying the vascular effects induced through flaxseed oil is not well known. Thus, in the present study, we investigated the effects of flaxseed oil on vascular function in isolated rat aortic rings.

**Methods:**

Wistar rats were treated daily with flaxseed oil or a control (mineral oil) intramuscular (i.m.) for fifteen days. Isolated aortic segments were used to evaluate cyclooxygenase-2 (COX-2) protein expression, superoxide anion levels and vascular reactivity experiments.

**Results:**

Flaxseed oil treatment increased the vasoconstrictor response of aortic rings to phenylephrine. Endothelium removal increased the response to phenylephrine in aortic segments isolated from both groups, but the effect was smaller in the treated group. L-NAME incubation similarly increased the phenylephrine response in segments from both groups. The TXA_2_ synthase inhibitor furegrelate, the selective COX-2 inhibitor NS 398, the TP receptor antagonist SQ 29.548, the reactive oxygen species (ROS) scavenger apocynin, the superoxide anion scavengers tiron and the phospholipase A_2_ inhibitor dexamethasone partially reversed the flaxseed oil-induced increase in reactivity to phenylephrine.

**Conclusions:**

These findings suggest that flaxseed oil treatment increased vascular reactivity to phenylephrine through an increase in ROS production and COX-2-derived TXA_2_ production. The results obtained in the present study provide new insight into the effects of flaxseed oil treatment (i.m.) on vascular function.

## Background

The consumption of flaxseed and its components has been associated with the prevention or reduction of cardiovascular disease. Flaxseed contains a mixture of fatty acids, primarily polyunsaturated fatty acids (PUFA), comprising 57% omega-3 α-linolenic acid (ALA), 16% omega-6 linoleic acid (LA) [1], and dietary fibers and phytoestrogen lignans [2].

The lignan in flaxseed possesses potent antioxidant effects [3], and PUFA have been associated with improvements in a variety of pathological conditions, such as myocardial infarction, atherosclerosis and hypertension [4-6]. Therefore, the cardiovascular effects of flaxseed could be attributed not only to PUFA, but also to the dietary fibers and phytoestrogen lignans presents in these grains. Previous reports concerning the vascular effect of flaxseed [7,8] have not provided evidence on whether the cardiovascular effects of flaxseed could be attributed to the PUFA present in the oil or the lignans and fibers present in the seeds.

We previously demonstrated that treatment with soybean oil for 15 days increases left ventricular performance without affecting arterial blood pressure in rats [9]. Considering the cardioprotective mechanisms induced through soybean oil [9], which is rich in LA, and other oils rich in PUFA, we investigated whether flaxseed oil treatment would produce vascular benefits. The effects of ALA on the cyclooxygenase-2 (COX-2) pathway have been shown to decrease thromboxane A_2_ (TXA_2_) and increase prostacyclin 3 (PGI_3_) in vessels, such as the aorta [10-12], which in turn, could augment endothelium-dependent vasodilatation. However, LA has been shown to increase series 2 prostaglandins and thromboxane, which increases vascular tone [13]. However, there is limited evidence regarding the vascular effects of flaxseed oil, which is rich in PUFA, such as ALA. Thus, the primary aim of the present study was to investigate the effects of flaxseed oil treatment on endothelium-dependent vascular reactivity.

As ALA is highly susceptible to oxidation, flaxseed oil could also induce lipid peroxidation [14,15], which might adversely effect the protection of the vascular system. Thus, a secondary aim of this study is to verify the influence of flaxseed oil treatment on reactive oxygen species (ROS) and COX-2 pathways.

## Results

**Figure 1:**
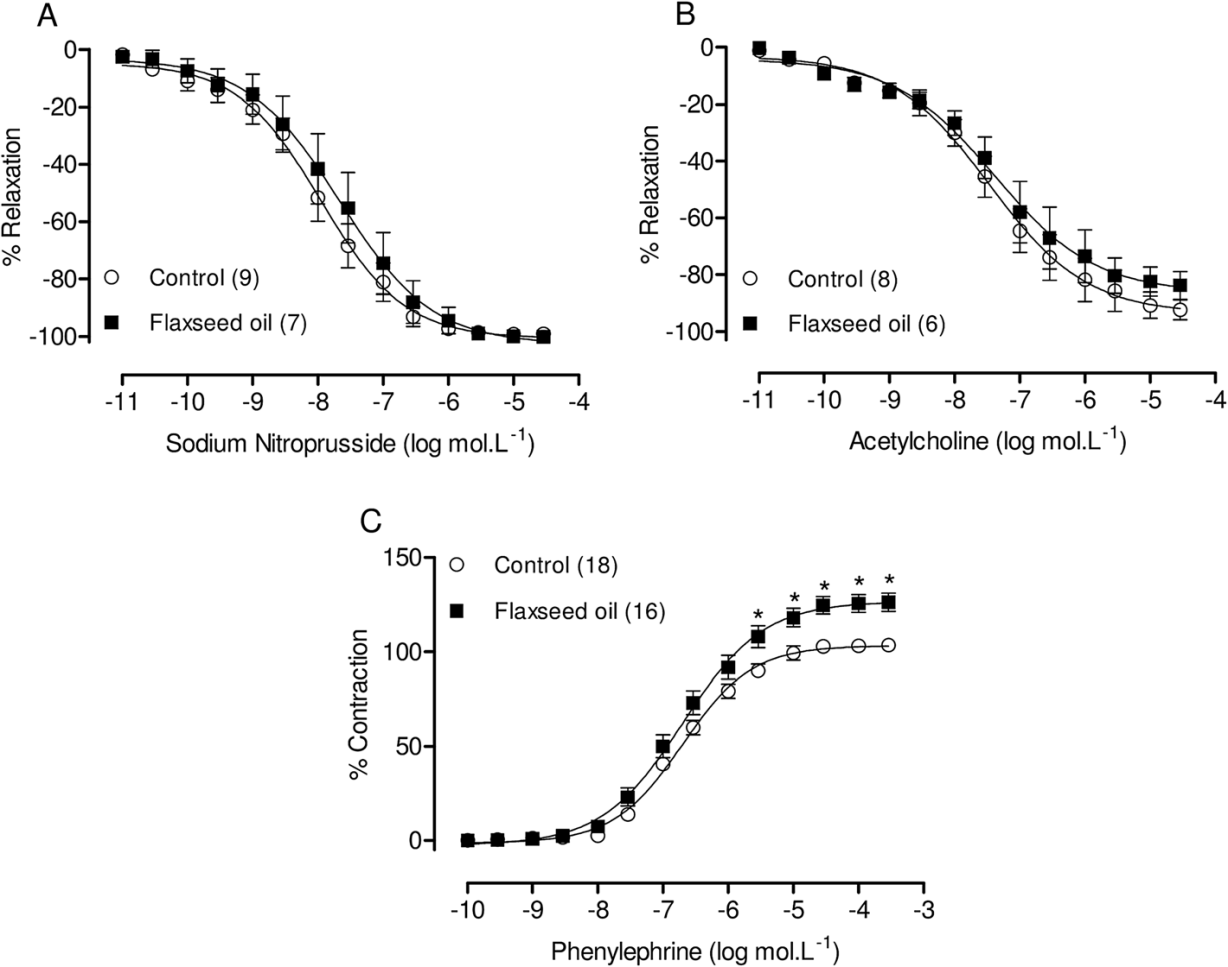
**Effect of flaxseed oil treatment on the vascular relaxation and vasoconstrictor response.** Concentration-response curves to sodium nitroprusside **(A)**, acetylcholine **(B)** and phenylephrine **(C)**. *P < 0.05. The number of animals used is indicated in parentheses.

**Figure 2:**
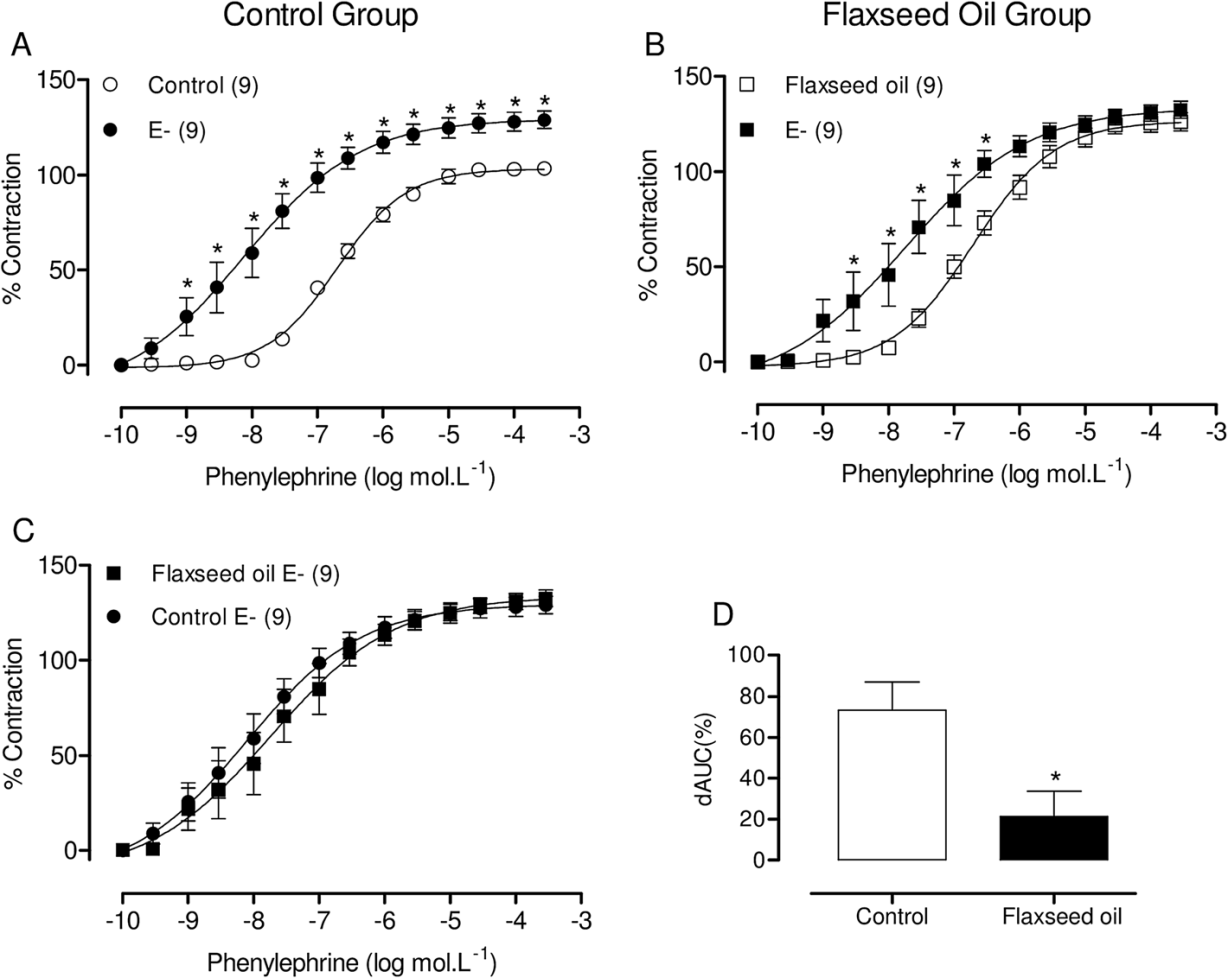
**Effect of flaxseed oil treatment on endothelial modulation of the vasoconstrictor response to phenylephrine.** Phenylephrine concentration-response curves after endothelium removal **(A, B, C)** in the aortic rings of control and flaxseed oil treated rats. E-: endothelium-denuded. Difference in the area under the concentration-response curves (dAUC) in endothelium–denuded and intact segments **(D)**. *P < 0.05. The number of animals used is indicated in parentheses.

**Figure 3:**
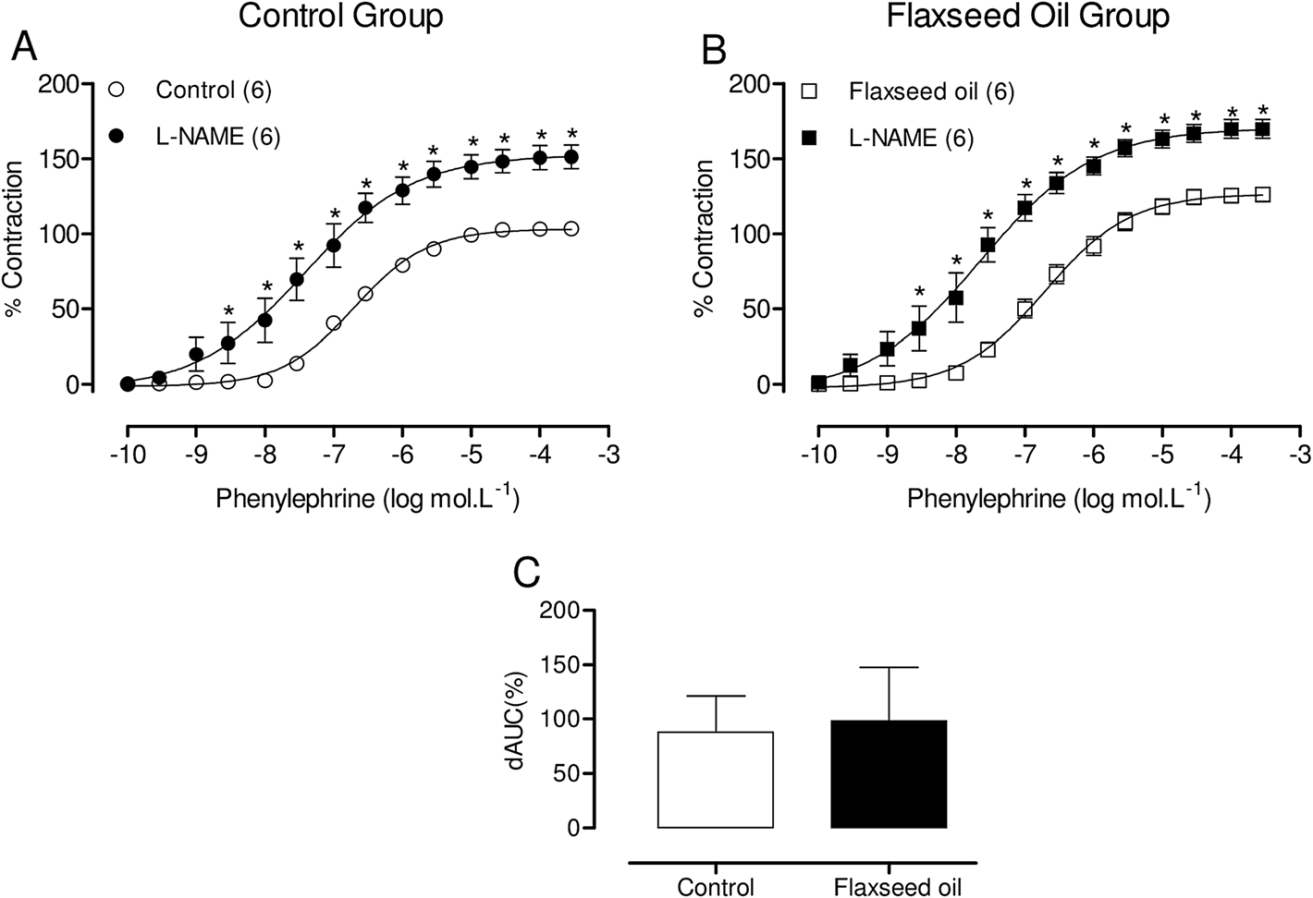
**Effect of flaxseed oil treatment on NO modulation of the vasoconstrictor response to phenylephrine.** Phenylephrine concentration-response curves after L-NAME incubation **(A, B)** in the aortic rings of control and flaxseed oil treated rats. Difference in the area under the concentration-response curves (dAUC) in the presence and absence of L-NAME **(C)**. *P <0.05. The number of animals used is indicated in parentheses.

No difference in body weight was observed between the groups either before (control = 250 ± 2.6 g, n = 21; flaxseed oil = 254 ± 2.47 g, n = 17; P > 0.05) or after (control = 314 ± 2.83 g, n = 21; flaxseed oil = 318 ± 2.16 g, n = 17; P > 0.05) treatment.Flaxseed oil treatment did not affect the response to KCl (control = 2.26 ± 0.14 g, n = 21; flaxseed oil = 2.13 ± 0.12 g, n = 17; P > 0.05) or the vasodilator responses induced through sodium nitroprusside or acetylcholine (Figure 1A and B, respectively) in the isolated aortic rings of treated rats compared with the controls. However, the maximal response to phenylephrine was increased in the aortic rings of flaxseed oil-treated rats, without modifying the sensitivity (control = 6.69 ± 0.06, n = 18; flaxseed oil = 6.68 ± 0.10, n = 16; P > 0.05) (Figure 1C).To evaluate the influence of the endothelium on the increased maximal response to phenylephrine, we mechanically removed the endothelium. Endothelium removal increased the response to phenylephrine in aortic segments isolated from both groups, but the effect was smaller in the treated group, as shown in Figure 2. This result suggests that flaxseed oil treatment reduces negative endothelial modulation.To evaluate whether flaxseed oil treatment alters NO modulation of the contractile responses induced through phenylephrine, the aortic rings were incubated with the nitric oxide synthase (NOS) inhibitor L-NAME (100 μM). L-NAME induced a similar left shift of the concentration response curve towards phenylephrine in aortic segments isolated from both groups and induced a similar contraction, as shown by the dAUC values (Figure 3). These findings suggest that flaxseed oil treatment did not affect NO production.

**Figure 4:**
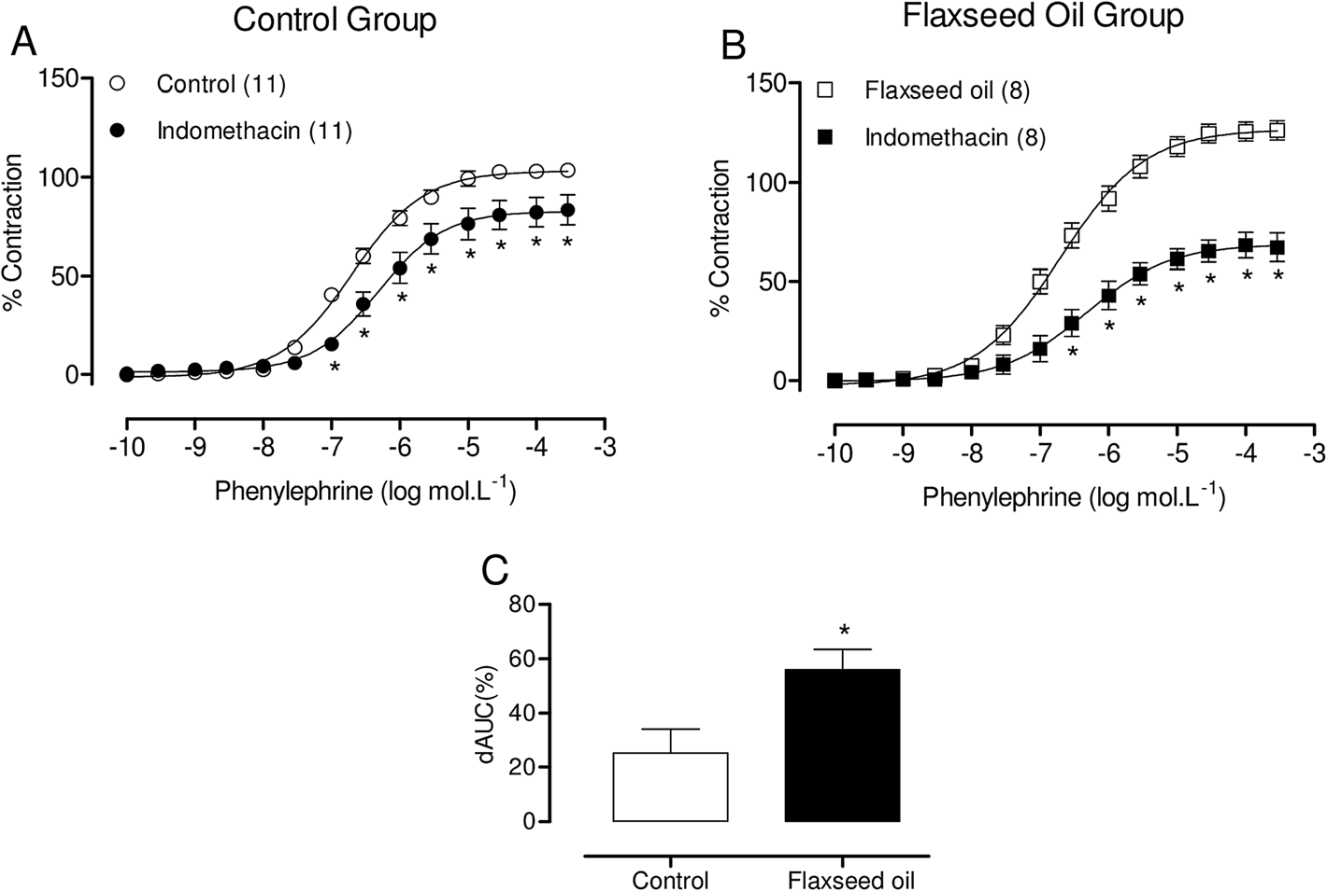
**Effect of flaxseed oil treatment on prostanoid modulation of the vasoconstrictor response to phenylephrine.** Phenylephrine concentration-response curves in the aortic rings of control and flaxseed oil-treated rats after indomethacin **(A, B)** incubation. Difference in the area under the concentration-response curves (dAUC) in the presence and absence of indomethacin **(C)**. *P <0.05. The number of animals used is indicated in parentheses.

Next, we investigated whether vasoconstrictor prostanoids played a role in the enhanced responses to phenylephrine in the aortic rings from flaxseed oil-treated rats using the non-selective cyclooxygenase inhibitor, indomethacin (10 μM). Indomethacin reduced the reactivity to phenylephrine in aortic rings from both groups, but the effect was greater in rings from the flaxseed oil-treated group, as shown by the dAUC values (Figure 4). These results suggest that vasoconstrictor prostanoids are involved in these responses. To clarify this issue, we investigated whether COX-2 or TXA_2_ influenced the effects of flaxseed oil treatment.

**Figure 5:**
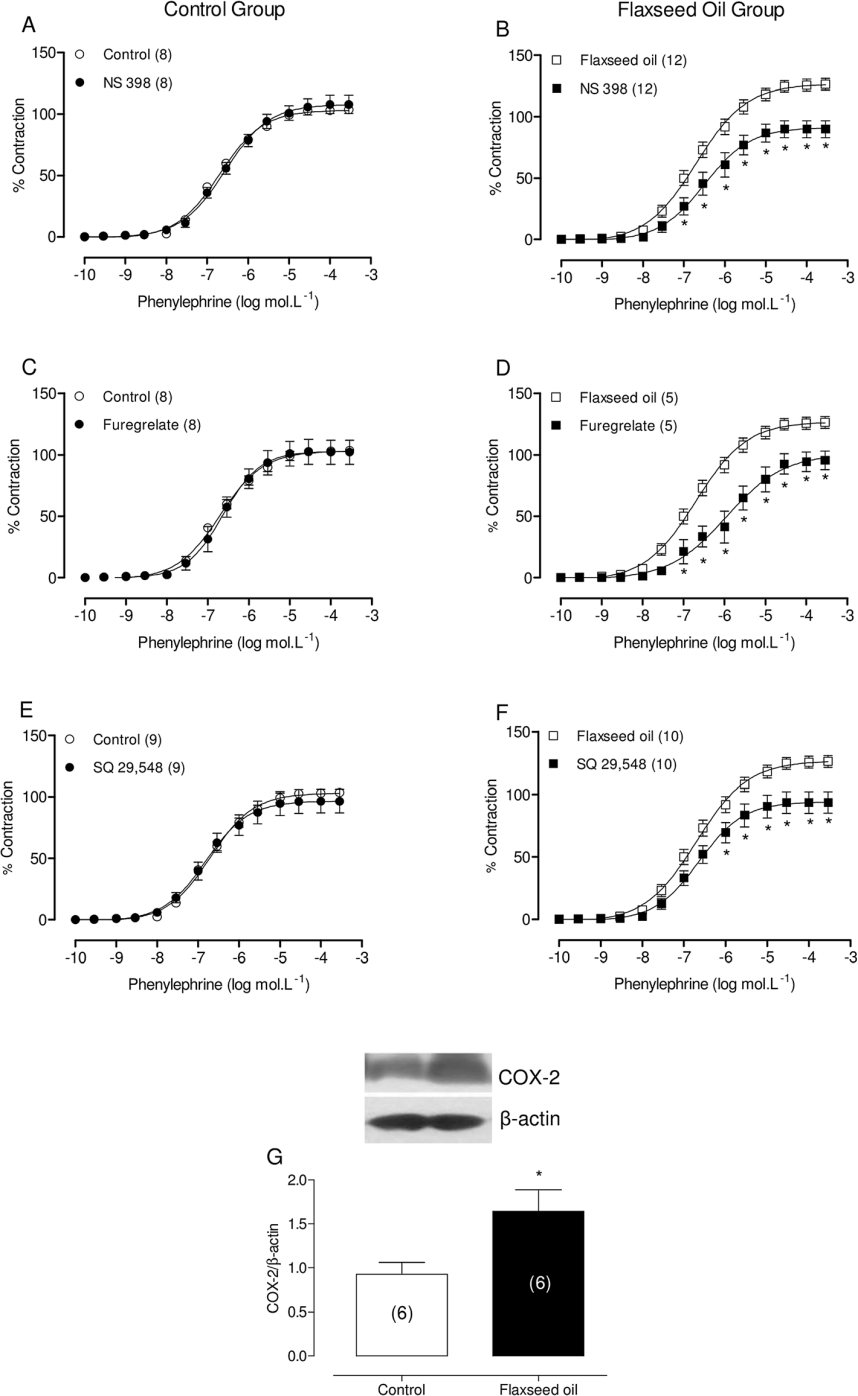
**Effect of flaxseed oil treatment on vasoconstrictor prostanoids modulation of the vasoconstrictor response to phenylephrine.** Phenylephrine concentration-response curves in the aortic rings of control and flaxseed oil-treated rats after NS 398 **(A, B)**, furegrelate **(C, D)** or SQ 29,548 **(E, F)** incubation. Densitometric analysis of the western blot for COX-2 **(G)**. Representative blots are also shown. *P <0.05. The number of animals used is indicated in parentheses.

The aortic rings were separately incubated with the COX-2 inhibitor NS 398 (1 μM), the TXA_2_ synthase inhibitor furegrelate (10 μM) or the receptor antagonist TP (TXA_2_ receptor - SQ 29.548, 1 μM). These inhibitors reduced vascular reactivity in all preparations from flaxseed oil-treated rats, but no alterations were observed in preparations from the control group (Figure 5A, B, C, D, E and F). We also examined COX-2 protein expression and observed higher expression in flaxseed oil-treated rats than in the controls (Figure 5G). These findings suggest that flaxseed oil treatment promotes vasoconstrictor prostanoid production through the COX-2 pathway, generating TXA_2_.

**Figure 6:**
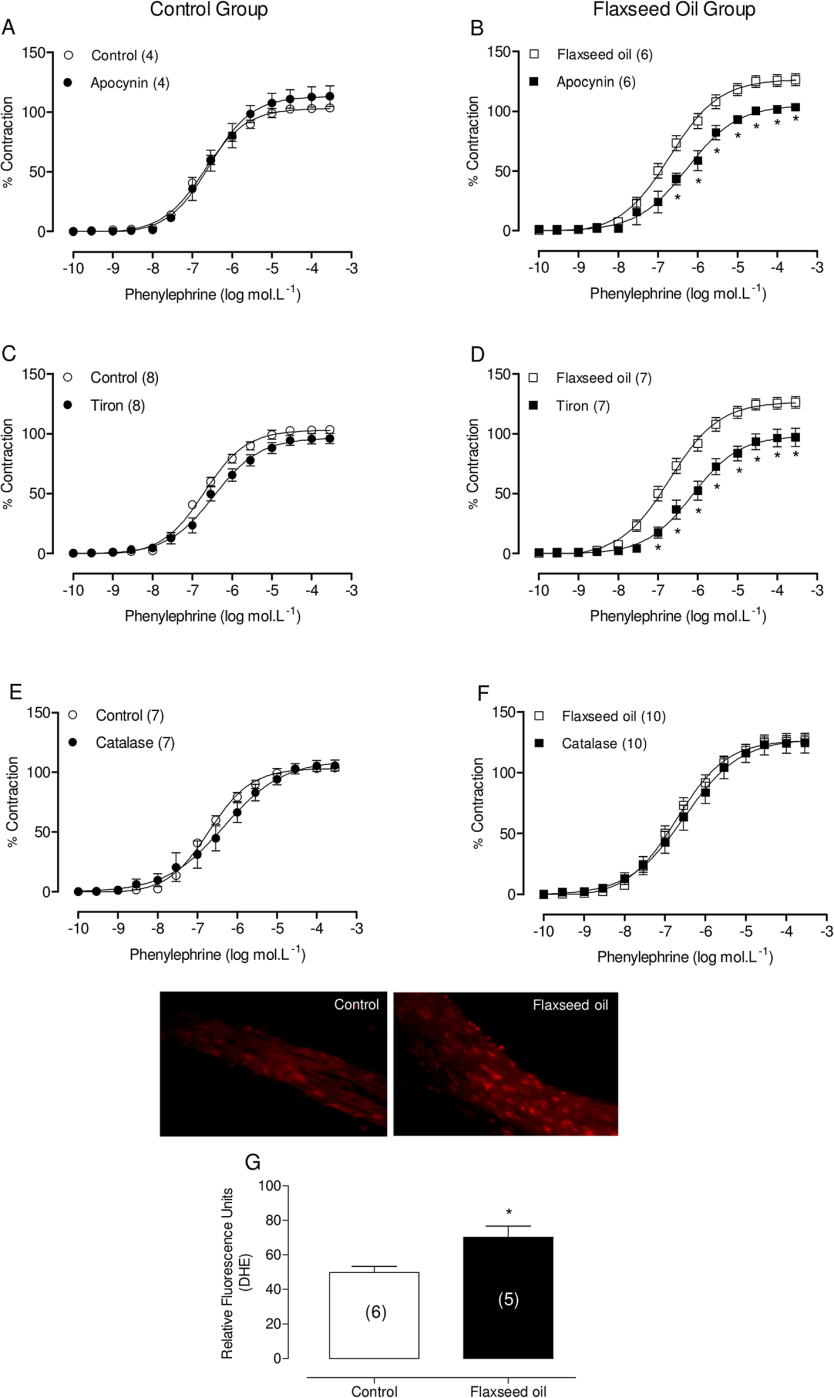
**Effect of flaxseed oil treatment on ROS modulation of the vasoconstrictor response to phenylephrine.** Phenylephrine concentration-response curves in the aortic rings of control and flaxseed oil-treated rats after apocynin **(A, B)**, tiron **(C, D)** or catalase **(E, F)** incubation. Vascular superoxide anion production in segments of aorta **(G)**. Upper: representative fluorescent photomicrographs of confocal microscopic arterial sections labeled with the oxidative dye hydroethidine. Below: vascular superoxide anion quantification. *P < 0.05. The number of animals used is indicated in parentheses.

In addition to COX-2-derived products, increased oxidative stress also promoted vascular hyperreactivity and endothelial dysfunction in the isolated aortic rings of treated rats. Therefore, the influence of ROS in the vascular response to phenylephrine after flaxseed oil treatment was evaluated using the ROS scavenger, apocynin (10 μM), the superoxide anion scavenger, tiron (1 mM), and the hydrogen peroxide scavenger, catalase (1000 U∙mL^−1^). Apocynin and tiron only reduced the vascular reactivity in aortic rings from flaxseed oil-treated rats (Figure 6A, B, C and D). However, catalase did not affect phenylephrine responses in any group (Figure 6E and F). In addition, we evaluated whether superoxide anion production is altered after flaxseed oil treatment. Figure 6G shows a significant increase in local superoxide anion production in the aortic rings from flaxseed oil-treated rats.

**Figure 7:**
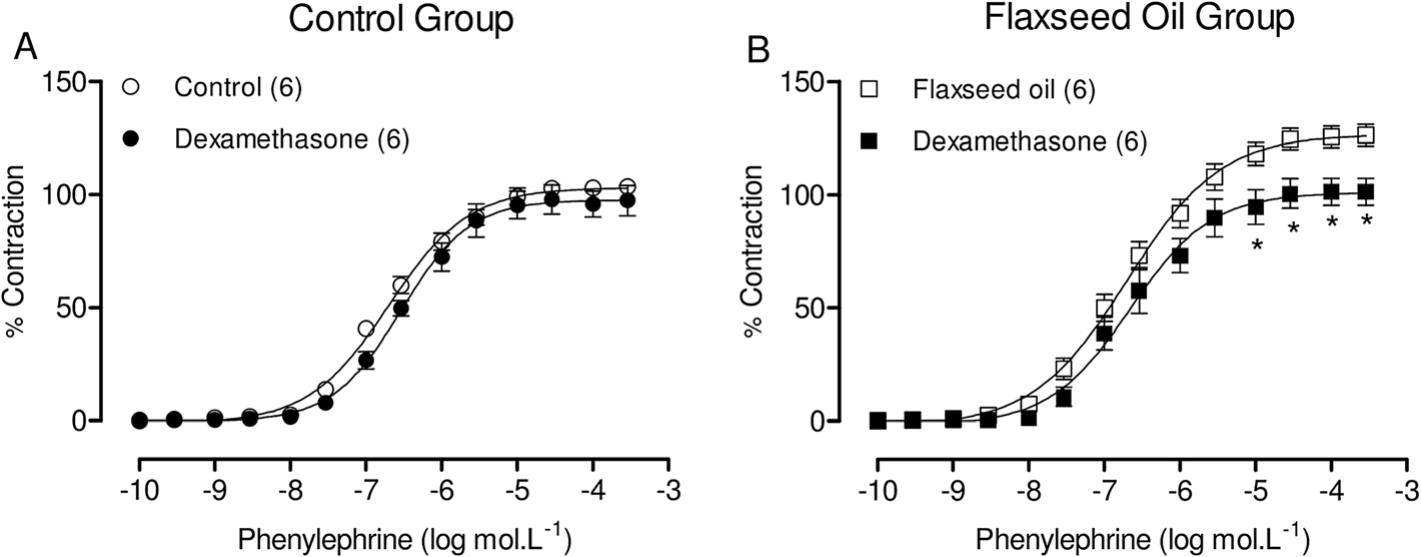
**Effect of flaxseed oil treatment on phospholipase A**_**2 **_**of the vasoconstrictor response to phenylephrine.** Phenylephrine concentration-response curves in the aortic rings of control and flaxseed oil-treated rats after dexamethasone **(A, B)** incubation. *P <0.05. The number of animals used is indicated in parentheses.

**Figure 8:**
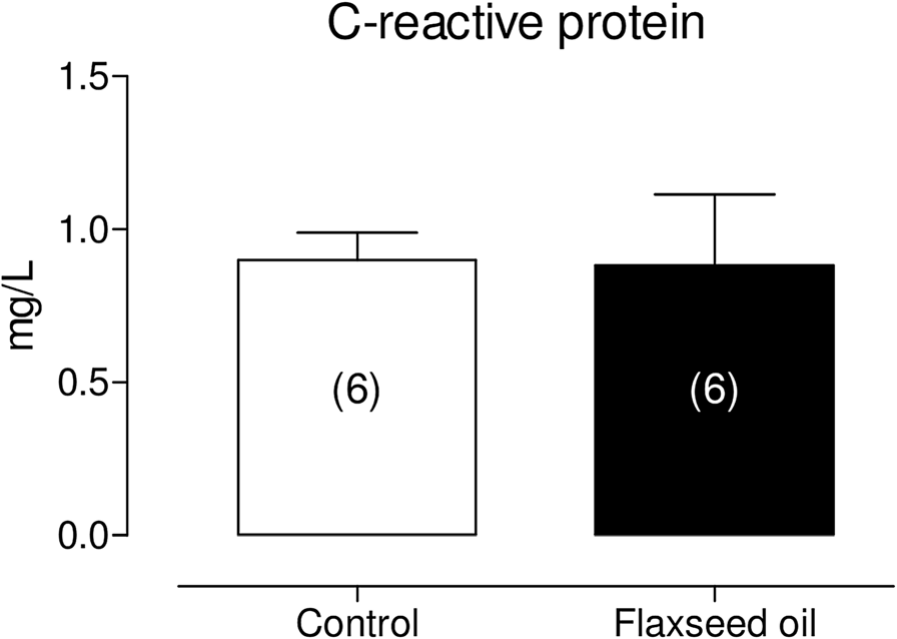
**Levels of C-reactive protein serum in control and flaxseed oil-treated rats.** P > 0.05. The number of animals used is indicated in parentheses.

Moreover, dexamethasone (1 μM) was used to investigate the putative role of phospholipase A_2_ on the increased vascular reactivity to phenylephrine induced through flaxseed oil treatment. The results showed that dexamethasone reduced the phenylephrine-induced contractile responses in isolated aortic rings from flaxseed oil-treated, but not control rats (Figure 7A and B).However, the effect of flaxseed oil treatment on inflammatory parameter determined by C-reactive protein, was not modified (Figure 8).

## Discussion

The present study is the first to show that treatment (i.m.) with flaxseed oil for two weeks increases the contractile response induced through phenylephrine in the rat aorta. This response is endothelium-dependent, and most likely reflects an increase of COX-2-derived TXA_2_ and superoxide anion production.

Flaxseed oil has recently attracted much research attention, as this seed is one of the richest vegetable oil sources of ALA, associated with cardiovascular benefits, and has lower amounts of LA; these compounds are derived from the omega-3 (C18:3 n-3) and omega-6 (C18:2 n-6) families, respectively [4-6,16].

Flaxseed oil contains n-3 ALA, a precursor molecule for the endogenous synthesis of f eicosapentaenoic acid (EPA) and docosapentaenoic acid (DHA) [17]. LA, which is present in lower amounts in flaxseed oil, is a precursor molecule for the synthesis of arachidonic acid (AA) [17]. Some reports have demonstrated that EPA and DHA prevent the development of hypertension [18,19] and exhibit anti-atherothrombogenic effects [20]. These effects might be induced, at least in part, through a decrease in TXA_2_ and an increase in PGI_3_ in vessels, such as the aorta [10-12]. Therefore, we investigated the effects of flaxseed oil treatment on endothelial modulation in the vascular responses induced through α-adrenoceptor activation in isolated aortic rings, as the aorta is a major artery susceptible to atherosclerosis.

In the present study, we observed increased vascular reactivity to phenylephrine in isolated aortic rings from flaxseed oil-treated rats. However, the treatment did not impair the endothelium-dependent relaxation induced through acetylcholine. Similarly, the vascular response to sodium nitroprusside was unaffected, suggesting that the endothelial-independent routes through which vascular relaxation is modulated were unaltered.

The effects of flaxseed on contractile and vasodilator responses are controversial. A previous report showed that feeding with a flaxseed-supplemented diet for 6, 8 or 16 weeks does not modify norepinephrine-induced vasoconstriction or the relaxation response induced through acetylcholine or sodium nitroprusside [7]. However, other reports have shown that a high-flaxseed diet enhances endothelial vasorelaxant function without reducing blood pressure in hypertensive rats [8]. Flaxseed is a rich source of plant lignans. The lignin secoisolariciresinol diglucoside in flaxseed possesses potent antioxidant effects [3]. Therefore, the improved endothelial vasodilatation, as previously demonstrated [8], could reflect the activities of lignans. However, Ogawa et al. [21] demonstrated that spontaneously hypertensive rats (SHR) fed a diet containing 10% flaxseed oil for four weeks did not show altered endothelium-dependent aortic relaxation in response to acetylcholine, although a significantly lower systolic blood pressure was detected.

To investigate the role of the endothelium in the increased phenylephrine responses induced through flaxseed oil treatment in aortic rings, the experiments in the present study were performed in the absence of endothelium. Endothelium removal promoted an increase in the phenylephrine-induced contraction in both groups, but this effect was smaller in the aortic rings from treated rats, suggesting that flaxseed oil affects endothelial function. These results suggest that the ability of the endothelium to negatively modulate the contractile response induced by phenylephrine may be impaired in isolated aortic rings of flaxseed oil-treated rats.

The increased vascular reactivity to phenylephrine and concomitant reduction of endothelial modulation in the aortic rings of treated rats might reflect reduced NO bioavailability [22]. Therefore, L-NAME was used to investigate the putative role of NO in the effects of flaxseed oil treatment on the contractile responses induced through phenylephrine. The results (Figure 3) showed that the phenylephrine responses were similar in the aortic rings of both control and treated rats, suggesting that the flaxseed oil treatment did not affect the endothelial-induced NO modulation of the vasoconstrictor response. The data concerning the effects of flaxseed oil on the nitric oxide pathway is limited and controversial. Sekine et al. [23] demonstrated that the oral administration of 1 mL flaxseed oil for 5 days reduces systolic blood pressure and increases prostaglandin I_2_ (PGI_2_) and NO release in SHR. However, Karaca and Eraslan [24] showed that 0.1 ml flaxseed oil administered through gavage for 30 days did not alter nitric oxide levels in the heart, brain and liver of rats.

Flaxseed oil is rich in ALA. ALA and LA are synthesized through a series of elongation and desaturation reactions and can subsequently be incorporated into cell membrane phospholipids [17]. EPA, DHA and AA act as alternative substrates for both cyclooxygenase (COX) and lipoxygenase (LOX) enzyme complexes, generating series 3 prostaglandins and thromboxanes and series 5 leukotrienes (EPA + DHA) or series 2 prostaglandins and thromboxanes and series 4 leukotrienes (AA) [25]. The series 2 prostaglandins and thromboxanes increase vascular tone. Therefore, to assess the participation of these compounds, we used indomethacin to assess whether vasoconstrictor prostanoids are involved in the increased vascular reactivity to phenylephrine induced through flaxseed oil treatment. Indomethacin reduced the response to phenylephrine in aortic segments from both groups, but this reduction was greater in the aortic rings of flaxseed oil-treated rats, suggesting the increased participation of the COX pathway. Moreover, NS 398, SQ 29.548 and furegrelate reduced the vasoconstrictor responses to phenylephrine in aortic segments from treated rats, suggesting that vasoconstrictor prostanoids, specifically TXA_2_, increase reactivity to phenylephrine induced through flaxseed oil treatment. Consistent with these results, we also observed that COX-2 protein expression was greater in flaxseed oil-treated rats than in control rats.

Tou et al. [26] demonstrated that female rats fed a diet supplemented with flaxseed oil for 8 weeks showed decreased liver AA, and no significant differences in the series 2 eicosanoids, PGE_2_ and TXB_2_ metabolites were observed. However, Lee et al. [27] showed that the aortic production of PGI_2_ and the serum concentration of TXA_2_ were significantly low in rats fed a diet containing flaxseed oil for 4 weeks. However, Rupp et al. [10] showed an increased production of 6-keto-PGF_lα_, a PGI_2_ by-product, in rats fed a diet supplemented with flaxseed oil, although the arachidonic acid content was greatly reduced. Sekine et al. [23] reported similar results, showing that the plasma 6-keto-PGF_lα_ levels were significantly higher in the fed flaxseed oil diet group.

PGI_2_ is synthesized in response to the release of the n-6 fatty acid AA from membrane phospholipids [25]. However, in addition to the increased PGI_2_, the n-6 LA could also increase other series 2 prostanoids, such as TXA_2_, a potent vasoconstrictor [25]. Moreover, a previous report demonstrated that high levels of PGI_2_ could induce a vasoconstrictor response through the activation of the TP receptor [28]. Although we did not measure levels of PGI_2_ in the present study, this hypothesis cannot be discarded.

Moreover, eicosanoids derived from PUFA, such as AA and EPA, are physiologically active compounds that act locally as signaling molecules through G protein-linked receptors. When AA is predominantly incorporated into cell membrane phospholipids under conditions, such as injury or inflammation, phospholipase A2 might release AA. Subsequently, intracellular signaling cascades might elicit a wide range of responses, including vasoconstriction, the activation of leukocytes, the stimulation of platelet aggregation and the generation of reactive oxygen species [29]. These effects might increase vulnerability to endothelial dysfunction and induce inflammation in the vessel wall, which is a key factor in atherosclerosis [30].

Thus, we suggest that the increased TXA_2_ induced through flaxseed oil treatment (i.m.) might increase phenylephrine contractions, as observed in the present study, through n-6 LA. Alterations in the eicosanoid profile might have important effects on inflammation [31]. The n-3 and n-6 PUFA compete for COX and LOX enzymes. Moreover, the series 2 and series 4 eicosanoids derived from n-6 PUFA are more biologically active than the series 3 and series 5 eicosanoids derived from n-3 PUFA [32], even when present in smaller amounts [33]. Furthermore, series 2 and series 4 eicosanoids can induce pro-inflammatory and pro-aggregatory states [34], likely promoting increased vascular reactivity [34].

Therefore, we used the phospholipase A_2_ blocker, dexamethasone, to investigate whether phospholipase A_2_ mediates the effects induced through flaxseed oil treatment in vascular reactivity. Dexamethasone abolished the effects of flaxseed oil on the contractile response to the α_1_-agonist, suggesting that a 15-day flaxseed oil treatment could increase phospholipase A_2_ activity, which in turn, increases eicosanoid production, leading to increased series 2 and series 4 eicosanoid activity. The effect of dexamethasone could be mediated through a reduction of COX-2-derived TXA_2_ release in response to α-adrenergic activation, as previously reported [35].

Glucocorticoids play an important role in the control of vascular smooth muscle tone through the modification of vasoconstrictor responses to different vasoactive agents and through alterations in vascular prostanoid and/or nitric oxide production [36,37]. Therefore, dexamethasone could inhibit COX-2 in the isolated aortic rings of flaxseed oil-treated rats, normalizing vascular reactivity to the control group level. In addition to COX-2-derived products, the increased oxidative stress could also promote vascular hyperreactivity and endothelial dysfunction in aortic rings isolated from treated rats. The results demonstrated that apocynin, a ROS scavenger, and tiron, a cell permeant non-enzymatic scavenger of O_2_^.–^, decreased vascular reactivity to phenylephrine in aortic rings isolated from treated rats, suggesting that flaxseed oil exposure increases ROS, primarily O_2_^.–^, and induces oxidative stress.

Consistent with these findings, we observed an increase of local superoxide anion production in the aortic rings from flaxseed oil-treated rats. However, incubation with catalase did not alter the vascular contractile responses in both groups, suggesting that flaxseed oil treatment did not affect the release of hydrogen peroxide. Martínez-Revelles et al. [38] demonstrated that COX-2-derived products modulate ROS production and also, ROS can activate COX expression. Thus, we suggest that COX-2 prostanoids might increase ROS production and ROS might act on COX-2 expression, although these COX-2-derived products and ROS could operate independently.

However, to investigate whether the flaxseed oil treatment could induce an inflammatory state, we evaluated serum C-reactive protein levels. Our results demonstrated that the flaxseed treatment did not modify this parameter. Flaxseed oil has variable effects on the inflammatory mediators that seem to be dose- and time- dependent. Lower doses do not affect TNF-a, IL-1b, IL-6, or soluble intracellular adhesion molecule-1 (sICAM-1) in healthy adults [39]. However, higher concentrations of flaxseed oil decrease the levels of cytokines [40]. Moreover, 4 months of flaxseed oil supplementation appears to reduce the C-reactive protein levels [41].

A previous report demonstrated that flaxseed oil treatment increases lipid peroxidation and oxidative stress and reduces SOD activity [42]. Another report showed that flaxseed oil does not affect catalase activity or superoxide dismutase in monkey livers *in vivo* and *in vitro*[43]. Although we did not measure SOD activity, these results suggest that flaxseed oil treatment increased ROS production, primarily the superoxide anion, without increasing hydrogen peroxide.

However, although the present study demonstrated increased ROS production, a recent study showed that the combined effect of flaxseed oil and astaxanthin, a natural antioxidant, in rats fed a high-fat diet efficiently ameliorates oxidative stress, lipid profile and inflammation [44].

However, as mentioned above, flaxseed oil was also reported to promote and increase lipid peroxidation [42]. Consistently, Nestel et al. [14] demonstrated that ALA reduces HDL and increases LDL plasmatic levels, likely reflecting high intake of ALA and increased vulnerability of polyunsaturated fatty acids to oxidation. This effect might alter the negative modulation of vascular tone in the endothelium, as shown in the present study.

In conclusion, the present study demonstrated that flaxseed oil treatment increases the vascular reactivity to phenylephrine and is associated with increased ROS production and the increased participation of COX-2 derivatives. Moreover, this treatment could increase phospholipase A_2_ participation, which in turn could increase the production of COX-2 inflammatory derivatives. Thus, the results of the present study provide new insights into the effects of flaxseed oil on vascular function.

## Materials and methods

### Animals and treatment

Male Wistar rats (250–300 g) were used in this study. The care and use of laboratory animals were in accordance with the Guide for the Care and Use of Laboratory Animals, and the protocols were approved through the Ethics Committee of the Federal University of Espirito Santo (038/2012 CEUA-UFES). During treatment, the rats were provided free access to tap water and fed standard chow *ad libitum*.

The rats were divided in two groups: flaxseed oil treatment or control. The treated group received a daily dose of 0.1 mL intramuscular (i.m.) of flaxseed oil for two weeks [9], and the control group received the same amount of mineral oil for the same time period. Notably, we used i.m. flaxseed oil injections to ensure that all rats received the same daily dose.

At the end of the treatment, the rats were anesthetized with urethane (1.2 g/kg, ip). The serum samples were collected and stored at −80°C until they were used to assess C-reactive protein. The thoracic aortas were carefully dissected, and the connective tissue was removed. For vascular reactivity experiments, the aortas were divided into cylindrical segments of 4 mm in length. To analyze the expression of COX-2 isoforms and evaluate O_2_^.–^ production in situ, the aortic segments were frozen at −80°C until the day of analysis.

### Vascular reactivity measurements

The aortic segments, 4 mm in length, were mounted in an organ bath at 37°C containing 5 mL Krebs-Henseleit solution (in mM: NaCl 118, KCl 4.7, NaHCO_3_ 25, CaCl_2_-2H_2_O 2.5, KH_2_PO_4_ 1.2, MgSO_4_–7H_2_O 1.2, glucose 11 and ethylenediamine-tetraacetic acid (EDTA) 0.01), continuously gassed with a 95% O_2_-5% CO_2_ mixture (pH 7.4). The arterial segments were stretched to an optimal resting tension of 1 g. The isometric tension was recorded using a force transducer (TSD125C, BIOPAC Systems, Santa Barbara, CA, USA) connected to an acquisition system (MP100, BIOPAC Systems).

After a 45-min equilibration period, all aortic rings were exposed twice to 75 mM KCl (30 min each), to examine the functional integrity of the samples and assess the maximal induced tension. Subsequently, the endothelial integrity was examined using acetylcholine (10 μM) on segments previously treated with phenylephrine (1 μM). A relaxation equal to or greater than 90% was indicative of functional integrity in the endothelium. After a washout period (30 min), increasing concentrations of phenylephrine (0.1 nM to 0.3 mM) were applied. A concentration-response curve to this agonist was obtained, and the maximal tension was measured upon reaching a plateau.

The influence of the endothelium on the response to phenylephrine was examined through the mechanistic removal of the tissue (rubbing the lumen with a needle). The absence of endothelium was confirmed based on the inability of 10 μM of acetylcholine to produce relaxation, and the functional integrity of the vessel was evaluated through KCl contraction. When the integrity of the vessel was damaged, the rings were discarded. The role of endothelium-derived vasoactive factors in the phenylephrine-elicited contractile response was examined. The effects of the following drugs were evaluated: the nonspecific NOS inhibitor N-nitro-L-arginine methyl ester (L-NAME, 100 μM), an inhibitor phospholipase A_2_ (dexamethasone, 1 μM), a non-selective cyclooxygenase inhibitor (indomethacin, 10 μM), a COX-2 inhibitor (NS 398, 1 μM), a TP receptor antagonist (SQ 29.548, 1 μM), a TXA_2_ synthase inhibitor (furegrelate, 1 μM), a ROS scavenger (apocynin, 10 μM), a superoxide anion scavenger (tiron, 1 mM) and a hydrogen peroxide scavenger (catalase, 1000 U.mL^−1^). These drugs were added at 30 min prior to the generation of phenylephrine concentration-response curves.

In another set of experiments, following the 45-min equilibration period, aortic rings were treated with phenylephrine (1 μM) until a plateau was reached (approximately 15 min), and concentration-response curves to acetylcholine (0.01 nM to 30 μM) or sodium nitroprusside (0.01 nM to 30 μM) were obtained for both groups.

### In situ detection of vascular O_2_^.–^ production

The oxidative fluorescent dye dihydroethidium (DHE) was used to evaluate O_2_^.–^production in situ, as previously described [45]. Hydroethidine freely permeates cells, and in the presence of O_2_^.–^, this compound is oxidized to ethidium bromide, which is trapped through DNA intercalation. Ethidium bromide is excited at 546 nm and emits light at 610 nm. The frozen tissue segments were dissected into 10-μm-thick sections and placed on a glass slide. The serial sections were equilibrated under identical conditions for 30 min at 37°C in Krebs-HEPES buffer (in mM: 130 NaCl, 5.6 KCl, 2 CaCl_2_, 0.24 MgCl_2_, 8.3 HEPES, and 11 glucose, pH 7.4). Fresh buffer containing DHE (2 μM) was applied topically to each tissue section. Subsequently, the sections were coverslipped and incubated for 30 min in a light-protected humidified chamber at 37°C, followed by imaging using an inverted fluorescence microscope Leica DM 2500 with a 40× objective and a Leica DFC 310 FX camera, using the same imaging settings for control and flaxseed oil-treated samples. The fluorescence was detected using a 568-nm long-pass filter. For quantification, eight frozen tissue segments per animal were sampled for each experimental condition and averaged. The mean fluorescence densities in the target region were calculated using ImageJ software.

### Western blot analysis of COX-2 expression

COX-2 protein expression was detected in homogenates from the aortic segments of control and flaxseed oil-treated rats, as previously described [46]. The proteins in the homogenates were separated through 10% SDS-PAGE and subsequently transferred to nitrocellulose membranes, followed by incubation with mouse monoclonal antibodies for COX-2 (1:200; Cayman Chemical; Ann Arbor, MI, USA). After washing, the membranes were incubated with an anti-mouse immunoglobulin antibody conjugated to horseradish peroxidase (1:5000, StressGen, Victoria, Canada). After thorough washing, the immunocomplexes were detected using an enhanced horseradish peroxidase/luminal chemiluminescence system (ECL Plus, Amersham International, Little Chalfont, UK) and film (Hyperfilm ECL - International). The signals on the immunoblot were quantified using the National Institutes of Health Image V1.56 software. The same membrane was used to detect β-actin expression using a mouse monoclonal antibody (1:5000, Sigma, USA).

### Measurement of C-reactive protein

The C-reactive protein concentrations were measured in serum simples by turbidimetry (Integra 4000), after immunoprecipitation with RCPLX (Roche/Hitachi cobas c) at the University Hospital Cassiano Antônio Moraes.

### Statistical analyses

The vasoconstrictor responses induced through phenylephrine were normalized to the contraction induced through 75 mM KCl and expressed as a percentage of this contraction. The vasodilator responses are expressed as a percentage of the previous phenylephrine contraction. For each concentration-response curve, the maximum effect (R_max_) and the concentration of the agonist that produced one-half of the R_max_ (EC_50_) were calculated using nonlinear regression analysis (GraphPad Prism Software, San Diego, CA). The sensitivity of the agonists is expressed as pD2 (−log EC_50_). To compare the effects of L-NAME, indomethacin and endothelium denudation on the contractile response to phenylephrine, the differences in the area under the concentration-response curves (dAUC) for phenylephrine under control and experimental conditions were calculated. The AUCs were calculated from the individual curve plots (GraphPad Prism Software), and the differences are expressed as the percentage of the AUC for the corresponding control. These values indicate differences in the magnitude of the effect of each treatment in the control and flaxseed oil-treated rats. The results are expressed as the means ± SEM of the number of rats studied, and the differences were analyzed using Student’s *t*-test and two-way ANOVA for comparisons between groups. When ANOVA showed a significant treatment effect, Bonferroni’s post hoc-test was used to compare individual means. Differences were considered statistically significant at P-values < 0.05.

### Drugs and reagents

Flaxseed oil and dexamethasone were purchased from Cacalia Comercial Ltda (natural products) and Alquimia Compounding Pharmacy, respectively. L-NAME, 1-phenylephrine hydrochloride, indomethacin, NS 398, SQ 29.548, furegrelate, acetylcholine chloride, sodium nitroprusside, apocynin, tiron and catalase were purchased from Sigma-Aldrich (St. Louis, USA). All salts and reagents were analytical grade and obtained from Sigma-Aldrich or Merck (Darmstadt, Germany).

## Abbreviations

PUFA: Polyunsaturated fatty acids; ALA: α-linolenic acid; LA: Linoleic acid; EPA: Eicosapentaenoic acid; DHA: Docosapentaenoic acid; i.m.: Intramuscular; COX: Cyclooxygenase; COX-2: Cyclooxygenase-2; TXA_2_: Thromboxane A_2_; PGI_3_: Prostacyclin 3; PGI_2_: Prostaglandin I_2_; AA: Arachidonic acid; LOX: Lipoxygenase; SHR: Spontaneously hypertensive rats; ROS: Reactive oxygen species; NO: Nitric oxide; NOS: Nitric oxide synthase; L-NAME: N-nitro-L-arginine methyl ester; DHE: Dihydroethidium; R_max_: Maximum effect; pD_2_: Sensitivity (concentration of the agonist that produced one-half of the R_max_); dAUC: Concentration-response curves.

## Competing interests

The authors declare that they have no competing interests.

## Authors’ contributions

DON was responsible for the data acquisition and analysis, manuscript preparation and discussion. CCPA, GBBF and MASCS contributed to the data acquisition and analysis and the discussion of the results. DVV, IS and ASP designed the experimental protocol, drafted the manuscript, participated in the discussion of the results and provided funding for the study. All authors read and approved the final version of the manuscript.
